# Novel utilization and quantification of Xsight diaphragm tracking for respiratory motion compensation in Cyberknife Synchrony treatment of liver tumors

**DOI:** 10.1002/acm2.14341

**Published:** 2024-04-15

**Authors:** Jianping Zhang, Lin Wang, Chenyu Xie, Zhiyu Yang, Benhua Xu, Xiaobo Li

**Affiliations:** ^1^ Department of Radiation Oncology Fujian Medical University Union Hospital Fuzhou China; ^2^ Fujian Medical University Union Clinical Medicine College Fujian Medical University Fuzhou China; ^3^ Department of Medical Imaging Technology College of Medical Imaging Fujian Medical University Fuzhou China; ^4^ Fujian Key Laboratory of Intelligent Imaging and Precision Radiotherapy for Tumors (Fujian Medical University) Fuzhou China; ^5^ Clinical Research Center for Radiology and Radiotherapy of Fujian Province (Digestive, Hematological and Breast Malignancies) Fuzhou China; ^6^ Department of Engineering Physics Tsinghua University Beijing China

**Keywords:** accumulated target coverage, liver tumor, respiratory motion compensation, Xsight diaphragm tracking

## Abstract

**Purpose:**

The Xsight lung tracking system (XLTS) utilizes an advanced image processing algorithm to precisely identify the position of a tumor and determine its location in orthogonal x‐ray images, instead of finding fiducials, thereby minimizing the risk of fiducial insertion‐related side effects. To assess and gauge the effectiveness of CyberKnife Synchrony in treating liver tumors located in close proximity to or within the diaphragm, we employed the Xsight diaphragm tracking system (XDTS), which was based on the XLTS.

**Methods:**

We looked back at the treatment logs of 11 patients (8/11 [XDTS], 3/11 [Fiducial‐based Target Tracking System‐FTTS]) who had liver tumors in close proximity to or within the diaphragm. And the results are compared with the patients who undergo the treatment of FTTS. The breathing data information was calculated as a rolling average to reduce the effect of irregular breathing. We tested the tracking accuracy with a dynamic phantom (18023‐A) on the basis of patient‐specific respiratory curve.

**Results:**

The average values for the XDTS and FTTS correlation errors were 1.38 ± 0.65  versus 1.50 ± 0.26 mm (superior‐inferior), 1.28 ± 0.48  versus 0.40 ± 0.09 mm (left‐right), and 0.96 ± 0.32  versus 0.47 ± 0.10 mm(anterior‐posterior), respectively. The prediction errors for two methods of 0.65 ± 0.16  versus 5.48 ± 3.33 mm in the S‐I direction, 0.34 ± 0.10  versus 1.41 ± 0.76 mm in the A‐P direction, and 0.22 ± 0.072  versus 1.22 ± 0.48 mm in the L‐R direction. The coverage rate of FTTS slightly less than that of XDTS, such as 96.53 ± 8.19% (FTTS) versus 98.03 ± 1.54 (XDTS). The prediction error, the motion amplitude, and the variation of the respiratory center phase were strongly related to each other. Especially, the higher the amplitude and the variation, the higher the prediction error.

**Conclusion:**

The diaphragm has the potential to serve as an alternative to gold fiducial markers for detecting liver tumors in close proximity or within it. We also found that we needed to reduce the motion amplitude and train the respiration of the patients during liver radiotherapy, as well as control and evaluate their breathing.

## 1 INTRODUCTION

Stereotactic ablation body radiotherapy (SABR) or stereotactic body radiotherapy (SBRT) for liver cancer has become increasingly favoured because of its encouraging success rate in controlling the disease and its well‐tolerated safety profile.^1^, ^2^, ^3^, ^4^, ^5^, ^6^, ^7^ The degree of potential diaphragmatic motion suggests that liver tumors can move considerably during respiration and diaphragmatic movement. Therefore, accurate delivery of the ablation dose to destroy tumor tissue while retaining as much of the healthy tissue intact as possible is a challenging task. Numerous motion monitoring devices have been employed to manage respiratory motion, including the Real‐Time Position Management (RPM) system, Response gating control,^8^ ANZAI gating system, abdominal compression,^9^ breath hold,^10^ and Synchrony Respiratory Tracking System (CyberKnife, Accuray, Inc., Sunnyvale, California, USA).^11^

The Synchrony respiratory tracking system, which includes both the fiducial‐based target tracking system (FTTS) and the Xsight lung tracking system (XLTS), effectively addresses respiratory motion by continuously monitoring and predicting the position of fiducial markers. This prediction is based on a correlation model derived from multiple sets of orthogonal x‐rays, which are subsequently linked to the positions of infrared light‐emitting diodes positioned on the chest wall. Patients can recline comfortably and breathe naturally during treatment, while the radiation beam dynamically adjusts its position in sync with the patient's respiratory movements. The major and minor complications associated with fiducial insertion for liver tumors can include coil migration, pneumothorax, bleeding, and death.^12^ Instead of depending on the recognition of fiducial markers in perpendicular x‐ray images, XLTS utilized a sophisticated image‐processing algorithm to accurately located the tumor and utilized triangulation for precise determination of its position, thus reducing the potential side effects associated with fiducial insertion.^13^ In addition, it eliminates invasive fiducial implantation procedures and reduces overall procedure time. Yang et al.^14^ conducted a study to explore the connection between the movements of a liver tumor and the diaphragm. Their findings suggested that diaphragmatic motion has the potential to serve as a dependable surrogate for monitoring liver tumor motion. Some published works have represented that the lung‐diaphragm border's information can be used as a surrogate during dynamic tumor tracking treatment. For example, Rostamzadeh et al.^15^ demonstrated the practicality of utilizing the diaphragm as a substitute for tracking liver targets on the Vero4DRT linear accelerator. Dick et al.^16^, ^17^ supplied the simulation study and validation study by using artificial neural networks (ANNs), and they showed the possibility of accurately predicting the tumor's location without relying on gold fiducial markers by relying on information from the lung‐diaphragm border has been demonstrated. Li et al.^18^ studied the respiratory synchronization tracking effect of Cyberknife stereotactic body radiotherapy with the diaphragm as the tracking target for lung cancer. However, no prior work exists on evaluates the practicality of employing the diaphragm as a substitute for liver SBRT treatment using CyberKnife.

According to the working principle of XLTS and the result of previous study, the diaphragm can function as a reliable substitute for monitoring liver tumors positioned within or in close proximity to the diaphragm, eliminating the need for placing gold fiducial markers in the liver. To our best understanding, this study represents a ground‐breaking advancement in the utilization of the diaphragm as an imaging tracking reference in an innovative XLTS for liver tumors situated within or in close proximity to the diaphragm. We refer to this novel method as the Xsight diaphragm tracking system (XDTS). In this study, our research focused on exploring the practicality of this innovative application using data from the CyberKnife motion tracking system (MTS) log files. And the results are compared with the patients who undergo the treatment of FTTS. The significance and uniqueness of our study can be outlined as follows:

1. Quantifying the correlation and prediction errors.
2. Conducting a tracking accuracy assessment by utilizing patient‐specific breath curves.
3. Investigating the target coverage during treatment.
4. Measuring the tumor's amplitude in three dimensions.
5. Evaluating respiratory baseline shifts and stability of respiratory center phase.
6. Analysing the relationships of the aforementioned evaluation indexes in all directions.

## 2 METHODS

### 2.1 Patient information

We chose eleven patients (8/11 [XDTS], 3/11 [FTTS]) with liver disease near or within the diaphragm, either primary or oligometastatic, in a retrospective manner. These patients were treated using SABR based on the novel XDTS or FTTS between 2017 and 2022. The patient details are provided in Table 1, and the approximate anatomical locations of their tumors are summarized and presented in Appendix 1a‐1h (XDTS) and Appendix 1i‐1k (FTTS).

**TABLE 1:** Patient characteristics.

Patient	Gender	Age	Diagnosis	Primary lesion	Tumor volumes (cm^3^)
Patient 1	Male	51		Hepatocellular carcinoma	224.15
Patient 2	Male	69		Hepatocellular carcinoma	20.53
Patient 3	Male	54	Metastasis	Esophageal carcinoma	9.31
Patient 4	Male	61	Metastasis	Rectal cancer	7.01
Patient 5	Male	68		Hepatocellular carcinoma	98.28
Patient 6	Male	57		Hepatocellular carcinoma	11.45
Patient 7	Male	72	Metastasis	Lung cancer	14.02
Patient 8	Male	59		Hepatocellular carcinoma	50.36

### 2.2 Respiratory tracking procedures and data collection

For all patients, the delivery plans were designed using the MultiPlan (V4.6) treatment planning system of CyberKnife, with tumor tracking carried out using XDTS and FTTS. Before the FTTS was used, minimum three fiducials were needed to track rotations, three to four fiducials were placed inside or near the liver tumor. The center of mass (COM) of fiducials was tracked and replaced the tumor when the delivery was performed. Because the liver tumor was hard to distinguish clearly in CT or x‐ray images. According to the principle of gold marker implantation from Accuray's user guide, the rule of fiducials for soft tissues was as follows. 1) Gold Seeds: Diameter: 0.7−1.2 mm, Length: 3−6 mm. 2) Place four to six fiducials following fiducial placement guidelines. 3) Minimum 20 mm spacing between fiducials in 3D space. 4) Triangle formed in 3D space by any three fiducials should have the smallest angle greater than 15°. 5) No more than 50–60 mm from target. 6) 20 × 20 cm Field of View for live images. 7) Ensure all fiducials can be visualized in 45° oblique views with no overlap. According to the principle of gold marker implantation above, the average distance between the center of mass (COM) of the markers and the tumor was approximately 2.9 cm. When the tracking method of XDTS was used, the tumor should be close to or in the diaphragm to minimize the correlation error of the model and enable general application. This was the vital step to build the tracking model. The diaphragm near the tumor was delineated as tracking tumor volume (TTV, represented by the blue region presented in the upper panel of Appendix 1a‐1h). It replaced the tumor and served as the foundation for creating the respiratory model when the plan was delivered. In this study, the mean COM between the TTV and tumor was 1.5 cm (< 2.9 cm). And this COM could be measured by MultiPlan TPS when the treatment plan was designed. This implied that the level of uncertainty in correlating the diaphragm with the actual movement of the tumor was similar to that of the Synchrony tracking technique. Throughout the treatment process, a set of orthogonal kV x‐ray images were taken at intervals ranging from 30 to 60 s. These images were used to validate the positions of the TTV or fiducials, and updated the tracking model. The treatment information generated from the MTS, including the tumor position, tracking errors, and external marker positions, was recorded in log files. We report the feasibility of XDT employing 2064 pairs of orthogonal kV x‐ray images and 2.7 million respiratory log data points acquired simultaneously during SBRT in eight patients afflicted by liver tumors, and presented the comparisons between two tracking methods (XDTS vs. FTTS). Detailed descriptions of the recorded logs were elaborated and explained as previously shown.^19^

### 2.3 Offline data evaluation

The tracking data for patients in the CyberKnife MTS log files were presented in four distinct coordinate systems: patient coordinates, image coordinates, robot coordinates, and camera coordinates. A rotation matrix was required during coordinate transformation, and its details are available elsewhere.^19^ In this study, all data were normalized to the patient coordinates.

### 2.4 Baseline flattening and rolling average calculation of motion data

**FIGURE 1:**
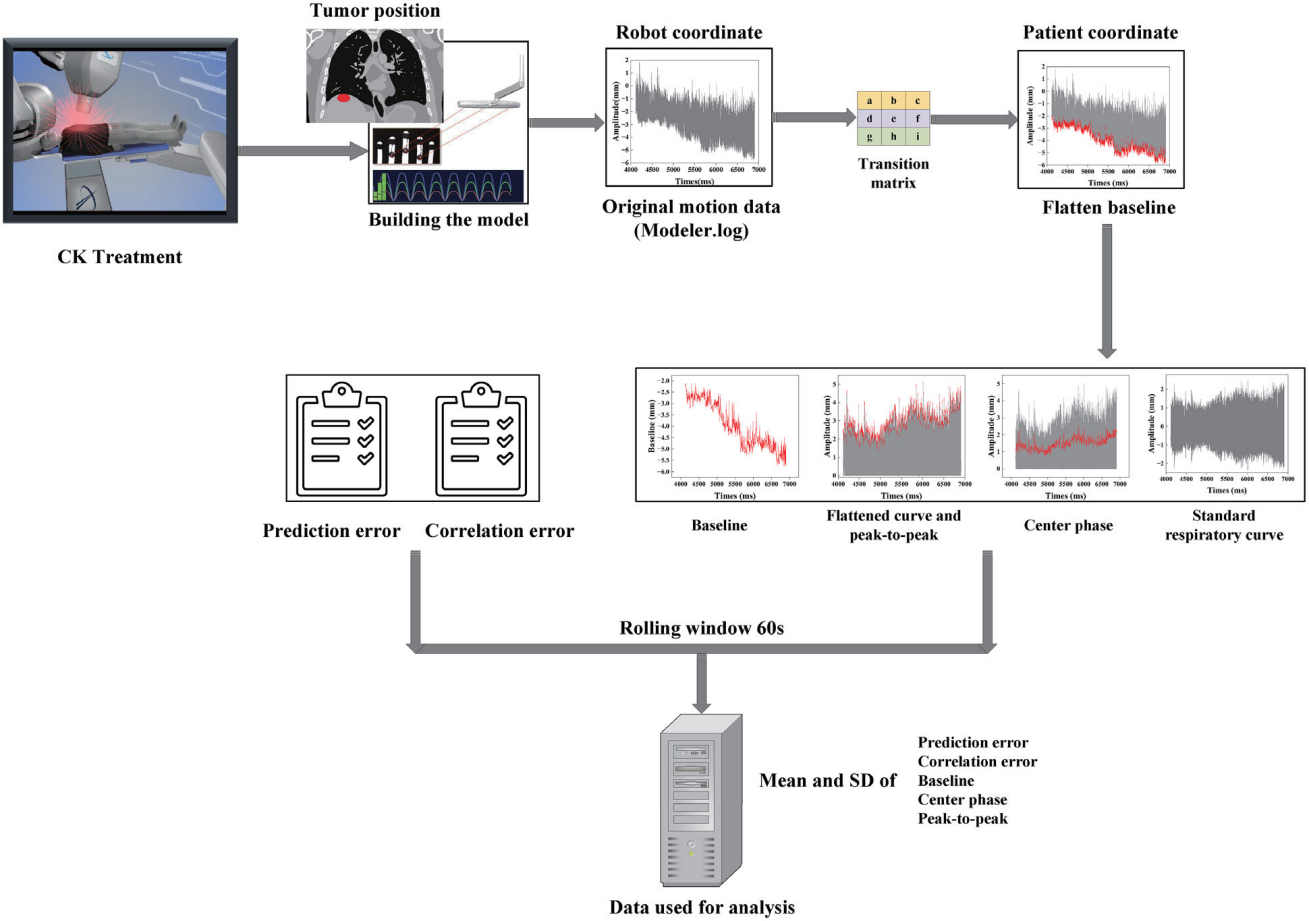
The data analysis method and workflow of the tracking accuracy of XDTS and FTTS for the liver tumor in CK synchrony treatment. Prior to the motion analysis, the baselines of the motion trace data were flattened to remove the portions with significant changes and improve the accuracy of the results. The respiratory data were calculated as a rolling average of the modeled tumor location in three directions to reduce the uncertainty of the effects of sudden irregular breathing.

Based on the patient tracking log data, we knew the baseline of the breath waveform would change because of irregular breathing patterns. Prior to motion analysis, the baselines of the motion trace data recorded in the Modeler log files were flattened to evaluate the baseline excursion of the tumor position and its influence on tracking accuracy, and to accurately calculate the amplitude of motion. However, the machine tracks the tumor using respiratory data based on the new baseline. Alternatively, calculations and analysis of the baseline, respiratory center phase stability, and motion amplitude would also be convenient. In order to reduce the effects of sudden and inconsistent breathing patterns, the respiratory information was smoothed by computing a moving average of the predicted tumor position. A 60‐s rolling window was established, which included several respiratory cycles and matched the duration required to capture the kV image sets for the modeling procedure. This indicates that the average values and deviations of the datasets, starting from D1 (the initial rolling window) to Dn, were computed by employing a time rolling window of 60 s. Figure 1 illustrated the data analysis methods and workflow used to analyse the tracking accuracy of XDTS and FTTS for liver tumors during the CyberKnife Synchrony treatment.

### 2.5 Tracking accuracy assessment by utilizing patient‐specific breath curves

Each patient may exhibit unique or distinct breathing patterns, and even the same patient may have different patterns for different fractions of their breaths. Researchers have reported on the tracking accuracy of the CyberKnife XLTS based on lung phantom by employing regular respiration curves such as sinusoids and curves collected from third‐party devices such as RPM and MR.^13^, ^20^, ^21^, ^22^, ^23^ However, what was rarely done was a retrospective analysis with the actual motion trace of each patient to run an E2E test. The actual breathing curve and tumor motion trajectory during treatment were first employed in our research to measure dynamic tracking uncertainty. In our study, the assessment of overall tracking accuracy, taking into account individualized patient respiratory patterns, was conducted through end‐to‐end (E2E) testing with a heterogeneous computerized imaging reference system (CIRS) phantom (Dynamic Thorax phantom, CIRS 18023‐A model, Norfolk, USA) on their respective ball‐cube inserts. The motion data was concurrently utilized to control two actuators: one for the tumor's trajectory and another for managing the motion of external optical markers. This process is described as follows.

1. Tumor motion trajectory curve. After the first minute of treatment, the standard tumor motion position data in three directions (removing the baseline drift) for the next 30 s were chosen as the tumor motion trajectory curve of the patient. A 95% confidence interval amplitude was computed using the 30‐s time‐step data.
2. Surrogate curve (external marker curve). Markers.log, recorded the three‐dimensional time‐stamped external optical markers. Depending on the placement of the Synchrony Camera Array, the Yc (y‐coordinate of the marker in the camera coordinate system) axis was nearly parallel to the Z axis of the patient coordinate system and often showed the most significant tracking marker motion along the Zp (z‐coordinate of the marker in the patient coordinate system, A‐P direction). As a result, the Yc values of the markers were derived from the log files and employed in the calculation of surrogate curves and amplitudes.
3. The two curves obtained from steps 1 and 2 were imported CIRS software.
4. Performing E2E targeting test. Dosimetry and beam‐targeting accuracy measurements were performed using a film insert with patient‐specific motion and surrogate curves using the CyberKnife system. The targeting errors from the film analysis for the XDTS E2E Test were then obtained.

### 2.6 Target coverage evaluation

**FIGURE 2:**
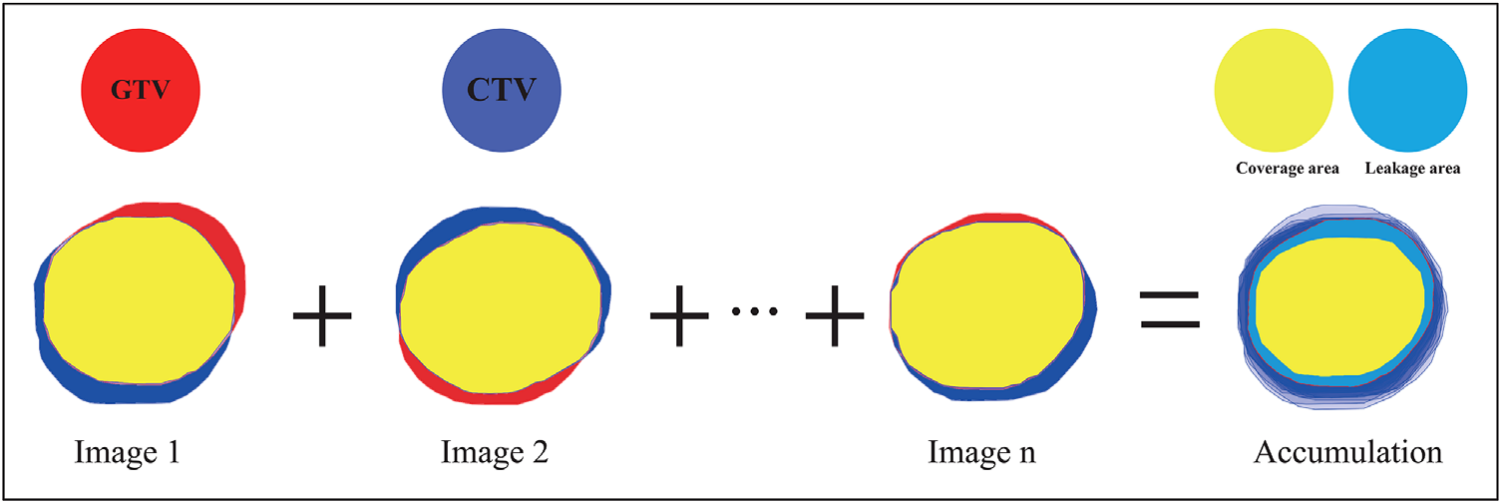
Example of coverage distribution within the planning target volume (PTV). For each control image acquired during treatment (1, 2,…, *n*), the intersection (yellow area) between the GTV (red structure) and the PTV (blue structure) was calculated and accumulated as target coverage.

In this analysis, we made the assumption that the deformation of the tumor was ignored. The tumor coverage was calculated to quantify the geometric treatment accuracy as follows: First, the actual tumor position center (gross tumor volume, GTV), which was organized according to the x‐ray imaging time‐step points and obtained from the ModelPoint log files documented in the patient coordinate system, was retrieved or extracted. Second, the predicted target [planning target position (PTV) or GTV] position center was logged into the prediction log file in robotic coordinates. Third, the target coverage of each voxel was determined by calculating the intersection of the GTV. The focus was on the precise location of the target, determined through live x‐ray imaging, which acted as the accurate reference point. And it also centered of the irradiated volume (e.g., PTV or GTV) per fraction per patient, as exemplified in Figure 2. To evaluate the effectiveness of the applied margin in covering the target volume, we utilized the standard that the coverage of 95% of the GTV should meet or exceed 95% (C95≥95%).

## 3 RESULTS

Appendix 2 showed the comparisons between XDTS and FTTS tracking methods, including correlation and prediction errors, target coverage, tumor amplitudes, baseline shifts, and stability of the respiratory center phase.

### 3.1 Correlation and prediction errors

**FIGURE 3:**
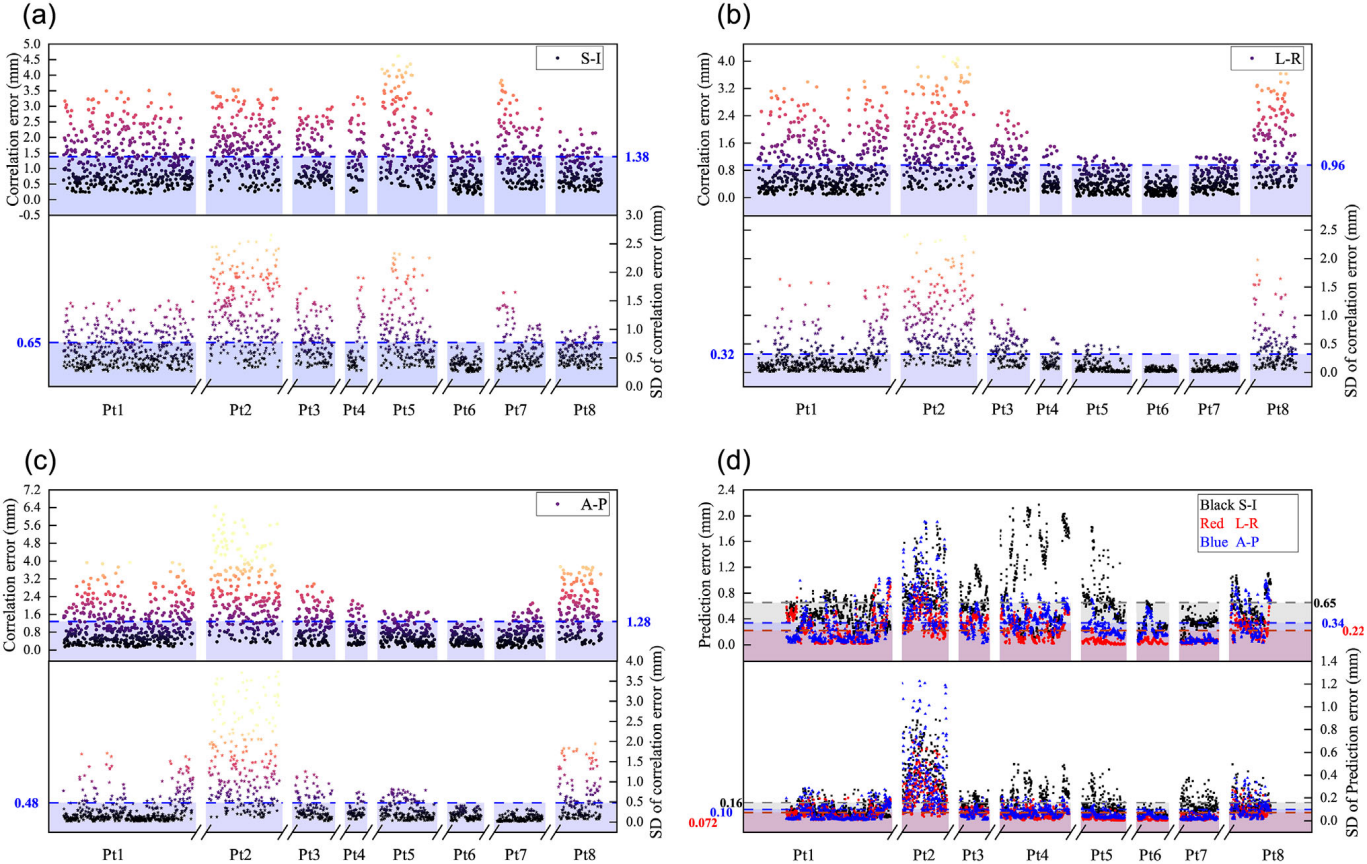
Correlation error (a)‐(c) and prediction error (d) for all patients in three directions. Note: The patient data were arranged in order from left to right (from fraction one to fraction *n*) as follows: Patient 1 (Fraction 1, …, *n*); Patient n (Fraction 1, …, *n*). The number was the mean value of all fractions from all patients.

Discrepancies between the model's predicted target positions and those obtained through x‐ray imaging were indicated by correlation errors. Figure 3a‐c displayed the distributions of correlation errors for each of the eight patients. The average values for the XDTS and FTTS correlation errors were 1.38 ± 0.65  versus 1.50 ± 0.26 mm (superior‐inferior), 1.28 ± 0.48  versus 0.40 ± 0.09 mm (left‐right), and 0.96 ± 0.32  versus 0.47 ± 0.10 mm(anterior‐posterior), respectively (see Appendix 2). In Figure 3d and Appendix 2, the modeled and predicted tumor positions had prediction errors for two methods of 0.65 ± 0.16  versus 5.48 ± 3.33 mm in the S‐I direction, 0.34 ± 0.10  versus 1.41 ± 0.76 mm in the A‐P direction, and 0.22 ± 0.072  versus 1.22 ± 0.48 mm in the L‐R direction.

### 3.2 Tracking accuracy assessment by utilizing patient‐specific breath curves

Table 2 listed the median (range) individualized curve‐based tracking accuracy for all patients. The differences between values for different patients was within 1 mm. The minimum or maximum accuracy value was used instead of two standard deviations from the mean. The largest variation between the centers of the planned and delivered doses remained within 1.5 mm, thereby meeting the requirements of the guide report of AAPM TG 135.^24^

**TABLE 2:** Targeting accuracy test based on patient‐specific curve for all patients.

Patient	Targeting accuracy (mm)
P1	0.98 (0.56–1.5)
P2	0.50 (0.26–1.0)
P3	0.61 (0.59–0.62)
P4	0.51 (0.25–1.21)
P5	0.91 (0.66–1.5)
P6	0.75 (0.71–1.49)
P7	0.66 (0.39–1.0)
P8	0.36 (0.27–1.4)

### 3.3 Target coverage during treatment

The target coverage per fraction per patient with five different margins (0 , 1 , 2 , 3 , and 5 mm) during the conventional FTTS and novel XDTS treatment is summarized in Table 3. The average target coverage of XDTS was 96.14% with a 2 mm margin and 98.03% with a 3 mm margin, respectively. While the values were 91.66 ± 15.63% (2 mm) and 96.53 ± 8.19% (3 mm) for FTTS, the average target coverage was larger than 95% with a 3 mm margin for two tracking methods.

**TABLE 3:** Target coverage of all patients with five different margins in the FTTS and novel XDT methods.

	0 mm	1 mm	2 mm	3 mm	5 mm
Pt1	92.88 ± 1.29	96.46 ± 1.17	98.27 ± 0.85	99.09 ± 0.51	99.71 ± 0.25
Pt2	79.46 ± 2.09	87.48 ± 2.35	93.20 ± 2.02	96.58 ± 1.10	99.32 ± 0.37
Pt3	76.62 ± 6.14	86.45 ± 6.29	92.46 ± 4.98	95.71 ± 3.05	98.62 ± 0.92
Pt4	81.27 ± 5.20	91.06 ± 5.06	95.99 ± 3.80	97.97 ± 2.69	99.40 ± 1.25
Pt5	90.52 ± 1.14	95.23 ± 1.07	97.66 ± 0.88	98.90 ± 0.64	99.73 ± 0.22
Pt6	89.12 ± 2.41	96.71 ± 1.76	99.43 ± 0.45	99.96 ± 0.05	100 ± 0
Pt7	83.89 ± 1.77	93.46 ± 1.90	98.18 ± 0.55	99.32 ± 0.52	99.95 ± 0.07
Pt8	82.26 ± 2.55	89.60 ± 2.46	93.94 ± 1.90	96.70 ± 1.40	99.25 ± 0.50
Total	84.50 ± 5.75	92.06 ± 4.01	96.14 ± 2.64	98.03 ± 1.54	99.50 ± 0.45

### 3.4 Tumor amplitudes in three directions

**FIGURE 4:**
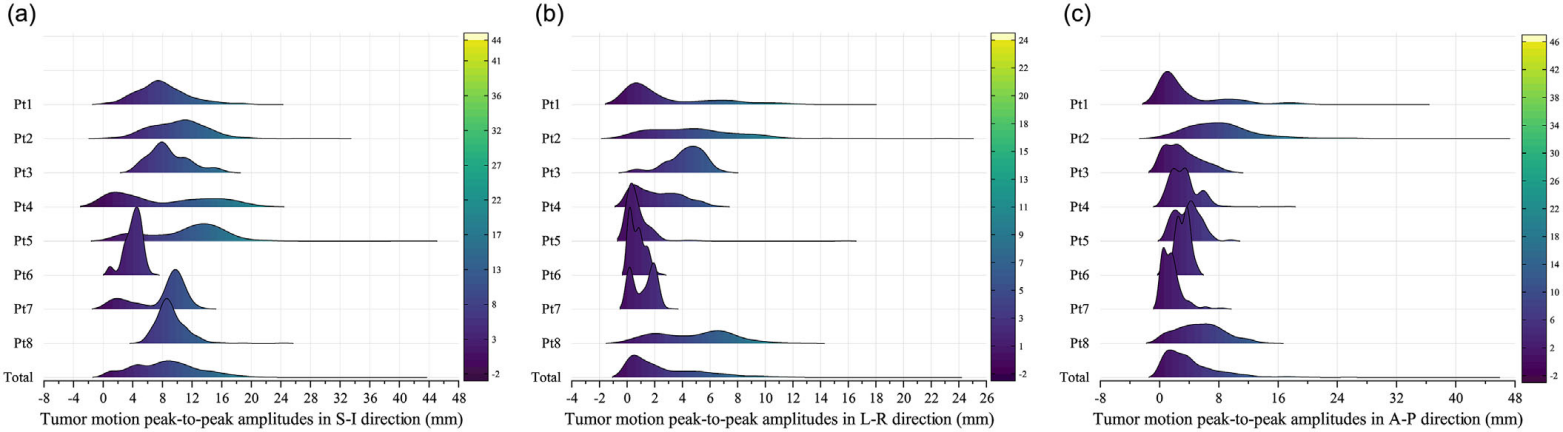
The peak‐to‐peak amplitude distribution of each patient for superior‐inferior (a), left‐right (b), and anterior‐posterior (c) components.

For every patient we treated, Figure 4 displayed the extent of movement (amplitude from peak to peak) in various directions. For the S‐I direction, the values had an average of 8.56 mm and a standard deviation of 4.54 mm. In the L‐R direction, the average was 2.77 mm with a standard deviation of 2.83 mm. Lastly, the A‐P direction had an average of 4.23 mm and a standard deviation of 3.92 mm. Some patients, like Patient 4 in the S‐I and A‐P directions, and Patient 7 in the S‐I and L‐R directions, exhibited dual peaks in the amplitude distribution. Considering the existence of two separate amplitude peaks, it is important to include an appropriate margin for the patient in the superior‐inferior (S‐I) direction.

### 3.5 Evaluating the respiratory baseline shifts and stability of the respiratory center phase

**FIGURE 5:**
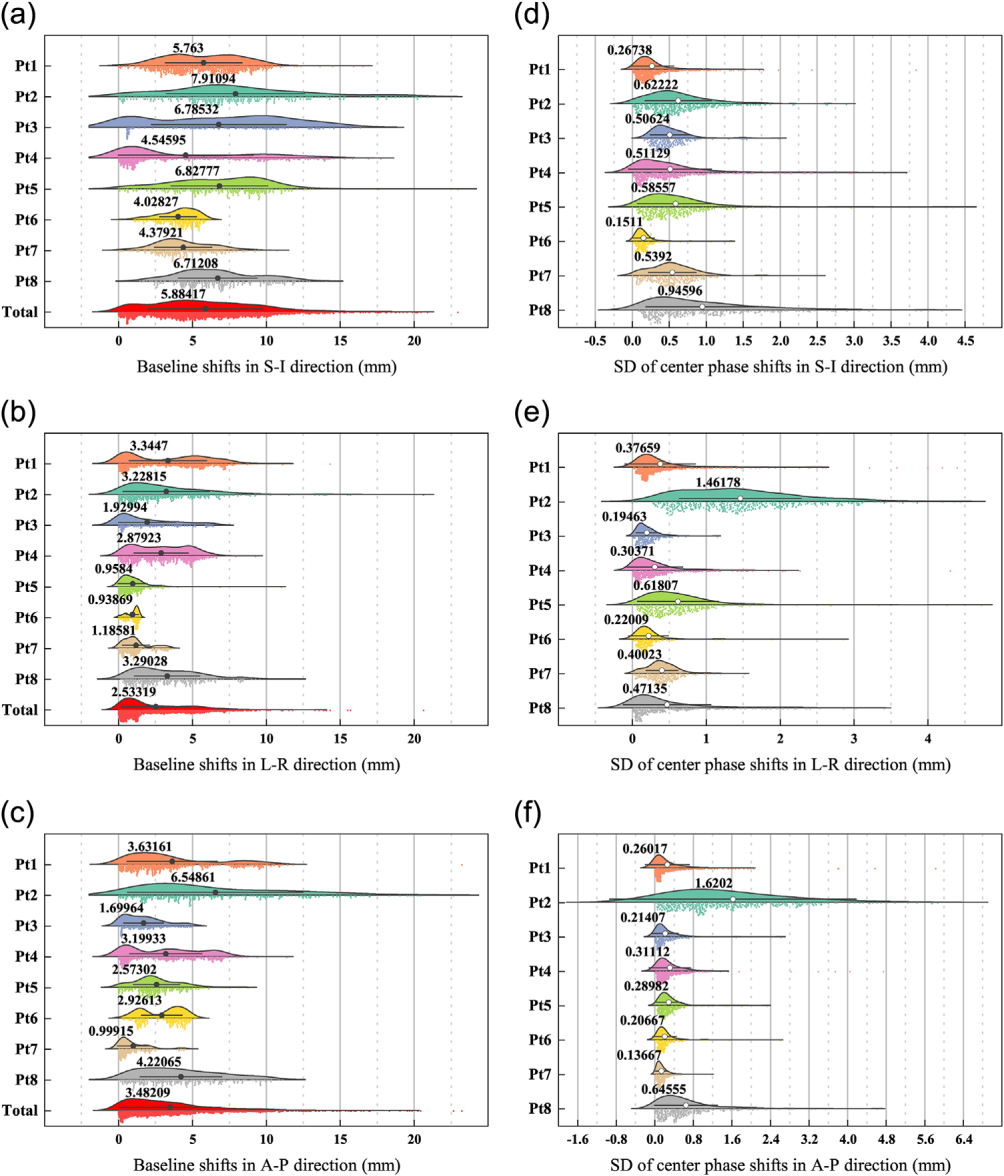
The baseline shift distribution of each patient in three directions (a)‐(c), and the standard deviations of center phase shift distribution of each patient in three directions (d)‐(f).

As shown in Figure 5a‐c and Appendix 2, the mean values of the total baseline shifts for XDTS were 5.88 mm for S‐I, 2.53 mm for L‐R, and 3.48 mm for A‐P, respectively. The results of FTTS were 6.97 mm (S‐I), 1.25 mm (L‐R), and 1.74 mm (A‐P). The standard deviations of the phase shift of the respiratory center were assessed for all patients and are shown in Figure 5d‐f. Except for Patient 2, the values in all directions were below 1 mm, and a bigger standard deviation resulted from more unstable motion or greater radial. However, as shown in Appendix 2, the average SD of center phase for FTTS were 3.63 ± 0.99 mm in S‐I, 0.55 ± 0.25 mm in L‐R, and 0.52 ± 0.24 mm in A‐P, respectively.

### 3.6 Relationships between the evaluation indexes

The correlation coefficient diagrams of the correlation and prediction errors, peak‐to‐peak amplitudes, baseline shifts, and SD of the respiratory center phase in all three directions were shown in Figure 6a‐c. The figure showed that the three components of XDTS have strong positive correlations with each other. These components were the peak‐to‐peak amplitude, the prediction error, and the SD of the respiratory center phase.

FIGURE 6The correlation coefficient diagrams among peak‐to‐peak amplitude, baseline shifts, SD of center phase, correlation error and prediction error in S‐I (a), L‐R (b) and A‐P, (c) directions, respectively.

**Figure:**
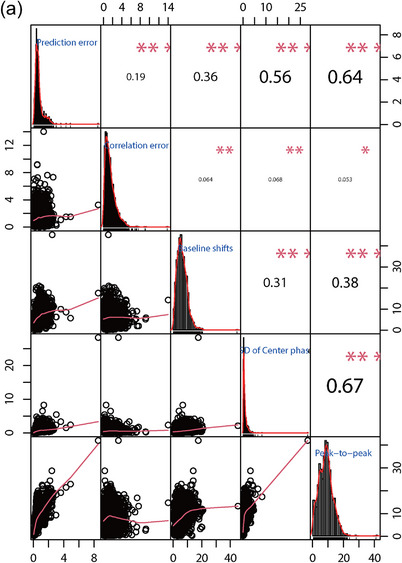

**Figure:**
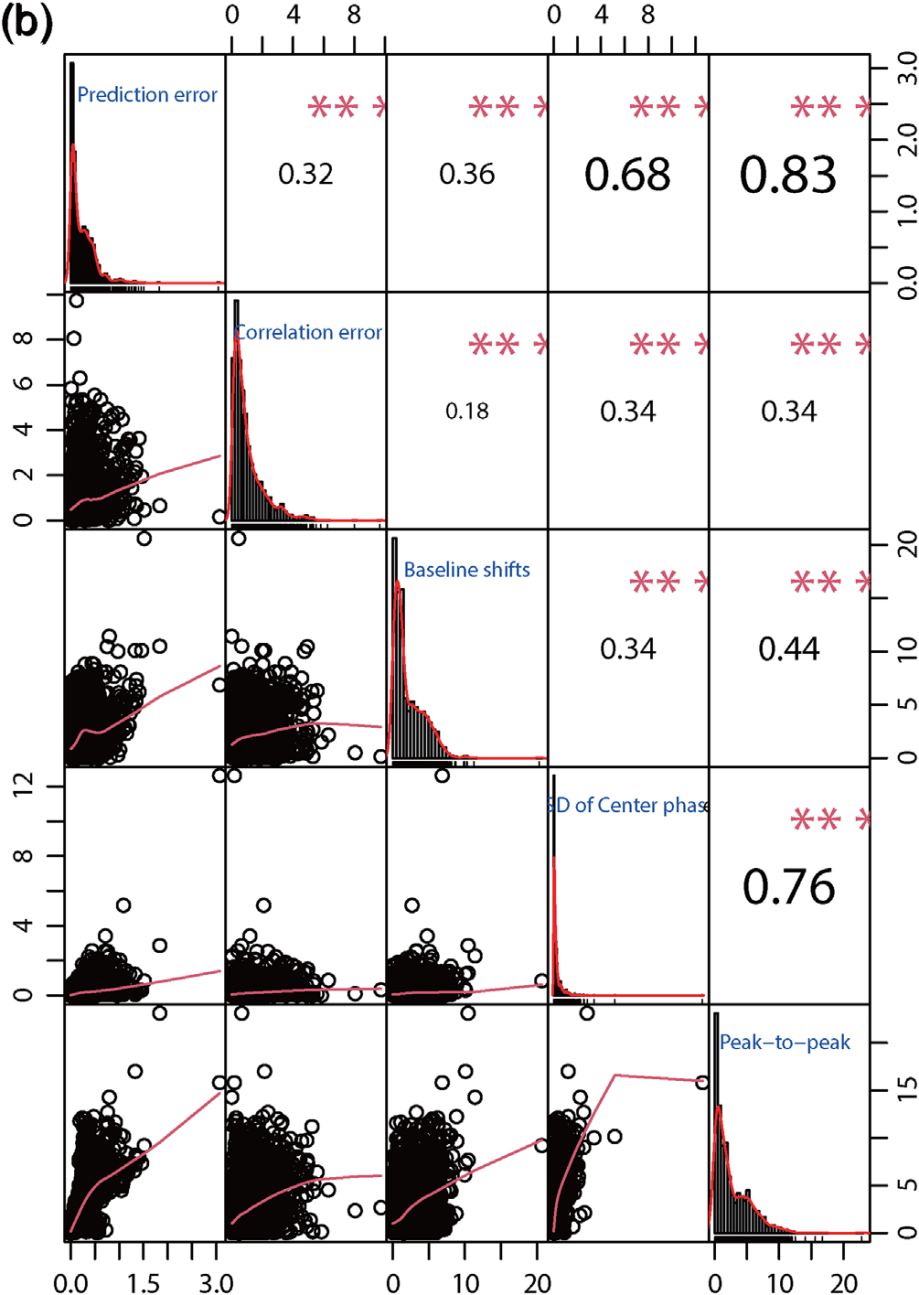

**Figure:**
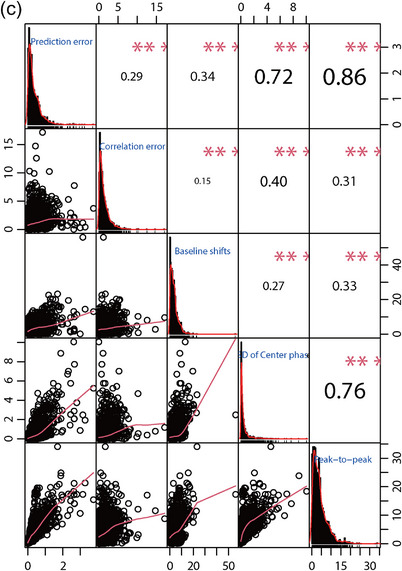

## 4 DISCUSSION

Several research studies have investigated the clinical efficacy and accuracy of the live respiratory monitoring system in CyberKnife, including the XLTS and the FTTS which is a target tracking system based on fiducials.^13^, ^22^, ^25^, ^26^, ^27^, ^28^, ^29^, ^30^ Their findings indicated that both the XLTS and the FTTS exhibited comparable performance with minor inaccuracies in monitoring the tumor's location throughout the treatment process. The CyberKnife real‐time respiratory tracking system has the capability to utilize either approach for administering precise radiation therapy to tumors that were affected by breathing movements. However, fiducials must be implanted into or near the tumor when FTTS is employed to deliver the ablation dose precisely and several complications are associated with fiducial insertion for liver tumors, such as coil migration, pneumothorax, bleeding, death. This is the first feasibility study to use the diaphragm as an imaging tracking volume in a novel XLTS for liver tumors situated within or in close proximity to the diaphragm, and we refer to this tracking method as XDTS.

The correlation errors of XDT calculated in our study were 1.38 ± 0.65 mm (S‐I), 1.28 ± 0.48 mm (L‐R), and 0.96 ± 0.32 mm (A‐P), respectively. According to Appendix 2, the correlation error of FTTS had a similar result compared with XDTS in S‐I direction. The slightly larger errors of XDTS were observed in L‐R and A‐P directions, while all the values were less than 1.5 mm. The results were consistent with those of Pepin et al.,^31^ where the correlation error differed in matching anatomic directions by approximately 1.5 mm. For XDTS, the S‐I direction had prediction errors of 0.65 ± 0.16 mm, the A‐P direction had prediction errors of 0.34 ± 0.10 mm, and the L‐R direction had prediction errors of 0.22 ± 0.072 mm. Zhang and colleagues^19^ reached a parallel conclusion, estimating prediction errors of 0.57 ± 0.32 , 0.01 ± 0.01 , and 0.20 ± 0.15 mm for the S‐I, L‐R, and A‐P directions, respectively, using fiducial‐based real‐time tracking. The prediction errors of FTTS were significantly larger than those of XDTS (see Appendix 2). The most probable reason for this phenomenon was that the fiducials migrated during the treatment course. Because the motion amplitude of diaphragm was significantly large, especially in S‐I direction. In terms of respiratory tracking accuracy, our results showed that XDTS for liver tumors had clinically acceptable tracking error (< 1.5 mm) compared with the standard fiducial‐based synchrony tracking. Therefore, this novel tracking method can be applied to liver tumors near the diaphragm without fiducial implementation.

This is the first study to discuss the accuracy of respiratory tracking using patient‐specific breathing curves and tumor motion trajectories. In our research, we initially employed the patient's breathing curve and tumor motion trajectory during treatment to quantify the dynamic tracking uncertainty. On Table 2, the median and maximum values for the precision of targeting were both less than 1  and 1.5 mm, correspondingly. Therefore, the tracking precision of the XDTS applied in this study is acceptable for clinical evaluation.

This study also evaluated the real‐time target coverage rate with different margins based on x‐ray images obtained during treatment. When the tumor expanded outward 2 mm, over 95% of the tumor volume received the recommended radiation dose, as stated in Section 3.3. Whereas the target coverage of patients 2, 3, and 8 were 93.2 ± 2.02%, 92.46 ± 4.98%, and 93.49 ± 1.90%, respectively. This phenomenon may be attributed to the wide range of breathing amplitudes in all three directions for these patients. According to the discussion of the correlation coefficient diagrams of Section 3.6, a larger breathing amplitude increases the prediction error and worsens the tracking accuracy. From Table 3, showed that the coverage rate of FTTS slightly less than that of XDTS. Therefore, a margin of 3 mm should be used to satisfy the clinical requirements based on the above results for both FTTS and XDTS tracking methods. Ricotti et al.^32^ proposed that the margin around the tumor volume should be 3 mm in all directions to ensure that at least 95% of the tumor volume receives the prescribed dose of radiation in the two‐view mode of XLTS. Therefore, first, patients with tumors near or inside the diaphragm were selected for this study, and the COM between the TTV and tumor was 1.5 cm compared to 2.9 cm obtained via the Synchrony tracking method. Second, as the diaphragm is located between the liver and lungs, using these two‐dimensional imaging features for motion tracking, such as XLTS was feasible. Third, the tracking accuracy and coverage rate met the clinical standards. This supported the notion that the diaphragm could function as a substitute for tracking liver tumors situated in or close to the diaphragm, eliminating the need for implanting gold fiducial markers in the liver. However, it is wise to investigate the margin on a patient basis using 4DCT.

In our study, the average (± standard deviation) tumor motion amplitudes calculated from the log files were 8.56 ± 4.54 mm (superior‐inferior), 2.77 ± 2.83 mm (left‐right), and 4.23 ± 3.92 mm (anterior‐posterior) for all XDTS tracking patients. This outcome aligned with the findings of Case et al.^33^ They measured the amplitude of liver motion, specifically the diaphragm's movement, through liver‐to‐liver alignment utilizing end‐exhale and end‐inhale CBCT as well as four‐dimensional CT reconstructions. They found that the mean liver motion amplitudes for all patients (range) were 1.8 (0.1−7.0), 8.0 (0.1−18.8), and 4.3 (0.1−12.1) mm in the (L‐R), (S‐I), and (A‐P) directions, respectively. The mean absolute interfraction diaphragm motion amplitude variations in our study were larger than the published mean values for the liver,^34^, ^35^, ^36^ which were 4.54 and 1.74 mm (S‐I), 2.83 and 0.34 mm (L‐R), and 3.92 and 0.93 mm (A‐P). Patients 1, 2, 5, 6, 7, and 8 exhibited a more concentrated amplitude distribution in all three directions than the other patients. Patient 4 exhibited two peaks in the amplitude distribution, which were specific characteristics of some patients (see Figure 4a‐c). However, the peak‐to‐peak amplitudes for three FTTS patients were 13.97 ± 1.89 mm in S‐I, 1.36 ± 0.27 mm in L‐R and 2.50 ± 0.46 mm in A‐P direction. As the discussion of the correlation coefficient diagrams, a larger breathing amplitude increased the prediction error and worsens the tracking accuracy. This was the reason why the tracking error, baseline shift, and SD of center phase of FTTS were worse than those of XDTS in S‐I direction (see Appendix 2). To obtain clinically acceptable coverage, we should be careful in adding a margin for patients for the S‐I and cases with different amplitude peaks.

The baseline shift is a critical factor to consider when assessing tumor motion, because the baseline shift may cause the tumor to receive less radiation than needed and the surrounding critical structures or normal tissue to receive more radiation than needed, especially for the cases with gating treatment. This study evaluated the real‐time liver tumor motion near the diaphragm by analysing the treatment data obtained from the CyberKnife system. In this study, we examined tumor excursions related to baseline shifts and variations in the respiratory center phase across treatment fractions. The average amount of the total baseline shift in the S‐I, L‐R, and A‐P directions was 5.88 , 2.53 , and 3.48 mm, respectively. The study by Sothmann et al.^3^ examined how the baseline drift affected the tracking accuracy of the treatment plan and detected small local dose variations of ± 3% inside the tumor. The study also used a γ‐evaluation method with a γ‐criteria of 1%/1 mm to compare the planned and delivered doses and found that 88% of the points agreed. In their study, Liang et al.^35^ observed a change in the initial position during liver SBRT using fiducial‐based real‐time tracking. They reported median values of 1.87 , 0.35 , and 1 mm for the baseline shift in the S‐I, L‐R, and A‐P directions, respectively. Therefore, more significant baseline fluctuations occurred in the motion traces when the liver tumors were situated in proximity to the diaphragm. Unstable peak changes in the L‐R and A‐P directions were observed in most patients, including Patients 1, 4, 6, 7, and 8, resulting in a baseline shift. Figure 5d‐f displayed the assessed standard deviations of the phase shift in the respiratory center for all patients. The overall values were below 1 mm in all directions, except for those of Patients 2, and greater unstable motion or larger radial resulted in a larger standard deviation. According to Appendix 2, the tracking error, baseline shift and stability of breathing amplitude were strongly proportional to the peak‐to‐peak amplitude. If the modeled and imaged results differ by too much (≥ 5 mm), the CyberKnife respiratory tracking treatment may be interrupted. Under these circumstances, the therapist may be required to re‐establish the model or adjust the treatment couch or patient position, which would prolong the treatment time and decrease the accuracy. These interrupted treatments had at least two causes: (1) the patient breathed irregularly or (2) the patient moved on purpose.

The correlation coefficient diagrams showed that the strong correlations between the five components corresponded to peak‐to‐peak amplitude and prediction error, SD of the center phase and prediction error, and SD of the center phase and peak‐to‐peak amplitude. According to Figure 6a‐c, the A‐P direction has the highest correlation coefficient, followed by the L‐R and S‐I directions. This means that reducing the peak‐to‐peak amplitude can improve the treatment accuracy more effectively.

## 5 CONCLUSION

This study investigated the possibility of compensating for respiratory motion using XDTS for liver tumors undergoing CyberKnife Synchrony treatment. The findings, based on targeting accuracy and target coverage rate, suggest that the diaphragm can effectively serve as a tracking reference for liver tumors situated in or near the diaphragm, obviating the need for gold fiducial markers in the liver. On the contrary, managing patient respiration, which includes training, control, evaluating breathing models in all directions, and reducing motion amplitude, proved essential when treating liver tumors with Stereotactic Ablative Body Radiotherapy (SABR).

## 6 LIMITATION

The motion data was generated every 38 ms by the MTS. Assuming a treatment time of 40 min for each patient at one fraction, there were over 60 000 data points related to respiration for every treatment fraction. More than 2.7 million log data points were analysed in this study. Hence, the findings of this study partially indicated the practicality of employing diaphragm tracking rather than fiducial markers for liver tumors situated in or close to the diaphragm.

## AUTHOR CONTRIBUTIONS

The manuscript was written by Jianping Zhang and Lin Wang, who also contributed to the conception, design, and interpretation of data. Chenyu Xie and Zhiyu Yang were responsible for the design and review of the treatment plans. Benhua Xu and Xiaobo Li performed the data analysis and review. All authors participated in the article writing and approved the final version.

Funding was provided by Fujian Province young and middle‐aged teacher education research project (JAT200165).

## CONFLICT OF INTEREST STATEMENT

The authors declare no conflicts of interest.

## Supporting information


**Appendix 1a**: The tumor anatomical locations for patient1.
**Appendix 1b**: The tumor anatomical locations for patient2.
**Appendix 1c**: The tumor anatomical locations for patient3.
**Appendix 1d**: The tumor anatomical locations for patient4.
**Appendix 1e**: The tumor anatomical locations for patient5.
**Appendix 1f**: The tumor anatomical locations for patient6.
**Appendix 1g**: The tumor anatomical locations for patient7.
**Appendix 1h**: The tumor anatomical locations for patient8.
**Appendix 1i**: The tumor anatomical locations for patient9.
**Appendix 1j**: The tumor anatomical locations for patient10.
**Appendix 1k**: The tumor anatomical locations for patient11.

Supporting Information

## References

[1] El Naqa I , Johansson A , Owen D , et al. Modeling of normal tissue complications using imaging and biomarkers after radiation therapy for hepatocellular carcinoma. Int J Radiat Oncol Biol Phys. 2018;100(2):335‐343. 29353652 10.1016/j.ijrobp.2017.10.005 PMC5779633

[2] Huang WY , Jen YM , Lee MS , et al. Stereotactic body radiation therapy in recurrent hepatocellular carcinoma. Int J Radiat Oncol Biol Phys. 2012;84(2):355‐361. 22342300 10.1016/j.ijrobp.2011.11.058

[3] Fast M , van de Schoot A , van de Lindt T , et al. Tumor trailing for liver SBRT on the MR‐Linac. Int J Radiat Oncol Biol Phys. 2019;103(2):468‐478. 30243573 10.1016/j.ijrobp.2018.09.011

[4] Hardy‐Abeloos C , Lazarev S , Ru M , et al. Safety and efficacy of liver stereotactic body radiation therapy for hepatocellular carcinoma after segmental transarterial radioembolization. Int J Radiat Oncol Biol Phys. 2019;105(5):968‐976. 31536781 10.1016/j.ijrobp.2019.09.006

[5] Kimura T , Takeda A , Tsurugai Y , et al. A multi‐institutional retrospective study of repeated stereotactic body radiation therapy for intrahepatic recurrent hepatocellular carcinoma. Int J Radiat Oncol Biol Phys. 2020;108(5):1265‐1275. 32712256 10.1016/j.ijrobp.2020.07.034

[6] Miften M , Vinogradskiy Y , Moiseenko V , et al. Radiation dose‐volume effects for liver SBRT. Int J Radiat Oncol Biol Phys. 2022;110(1):196‐205. 10.1016/j.ijrobp.2017.12.290 PMC6095822 29482870

[7] Ohri N , Tomé WA , Romero AM , et al. Local control after stereotactic body radiation therapy for liver tumors. Int J Radiat Oncol Biol Phys. 2022;110(1):188‐195. 10.1016/j.ijrobp.2017.12.288 PMC6102100 29395629

[8] Wong JW , Sharpe MB , Jaffray DA , et al. The use of active breathing control (ABC) to reduce margin for breathing motion. Int J Radiat Oncol Biol Phys. 1999;44(4):911‐919. 10386650 10.1016/s0360-3016(99)00056-5

[9] Eccles CL , Dawson LA , Moseley JL , et al. Interfraction liver shape variability and impact on GTV position during liver stereotactic radiotherapy using abdominal compression. Int J Radiat Oncol Biol Phys. 2011;80(3):938‐946. 20947263 10.1016/j.ijrobp.2010.08.003 PMC3037422

[10] Mageras GS , Yorke E . Deep inspiration breath hold and respiratory gating strategies for reducing organ motion in radiation treatment. Semin Radiat Oncol. 2004;14(1):65‐75. 14752734 10.1053/j.semradonc.2003.10.009

[11] Kilby W , Dooley JR , Kuduvalli G , et al. The CyberKnife Robotic Radiosurgery System in 2010. Technol Cancer Res Treat. 2010;9(5):433‐452. 20815415 10.1177/153303461000900502

[12] Ohta K , Shimohira M , Murai T , et al. Percutaneous fiducial marker placement prior to stereotactic body radiotherapy for malignant liver tumors: an initial experience. J Radiat Res. 2016;57(2):174‐177. 26826200 10.1093/jrr/rrv099 PMC4795956

[13] Jung J , Song SY , Yoon SM , et al. Verification of accuracy of CyberKnife tumor‐tracking radiation therapy using patient‐specific lung phantoms. Int J Radiat Oncol Biol Phys. 2015;92(4):745‐753. 25936598 10.1016/j.ijrobp.2015.02.055

[14] Yang J , Cai J , Wang H , et al. Is diaphragm motion a good surrogate for liver tumor motion? Int J Radiat Oncol Biol Phys. 2014;90(4):952‐958. 25223297 10.1016/j.ijrobp.2014.07.028 PMC4334361

[15] Rostamzadeh M , Thomas S , Camborde ML , et al. Markerless dynamic tumor tracking (MDTT) radiotherapy using diaphragm as a surrogate for liver targets. J Appl Clin Med Phys. 2023. doi:10.1002/acm2.14161:e14161 PMC10860457 37789572

[16] Dick D , Wu X , Hatoum GF , Zhao W . A fiducial‐less tracking method for radiation therapy of liver tumors by diaphragm disparity analysis part 1: simulation study using machine learning through artificial neural network. J Radiat Oncol. 2018;7(3):275‐284.

[17] Dick D , Wu X , Hatoum GF , Zhao W . A fiducial‐less tracking method for radiation therapy of liver tumors by diaphragm disparity analysis part 2: validation study by using clinical data. J Radiat Oncol. 2018;7(4):345‐356.

[18] Li GQ , Yang J , Wang Y , et al. Using the diaphragm as a tracking surrogate in CyberKnife synchrony treatment. Med Sci Monit. 2021;27:e930139. 34379616 10.12659/MSM.930139 PMC8366302

[19] Zhang J , Wang L , Li X , et al. Quantification of intrafraction and interfraction tumor motion amplitude and prediction error for different liver tumor trajectories in Cyberknife synchrony tracking. Int J Radiat Oncol Biol Phys. 2021;109(5):1588‐1605. 33227440 10.1016/j.ijrobp.2020.11.036

[20] Fu D , Kahn R , Wang B . Xsight lung tracking system: a fiducial‐less method for respiratory motion tracking. In: Urschel HC , Kresl JJ , Luketich JD , Papiez L , Timmerman RD , Schulz RA , eds. Treating Tumors that Move with Respiration. Springer Berlin Heidelberg; 2007:265‐282. doi:10.1007/978-3-540-69886-9_26

[21] Muacevic A , Drexler C , Wowra B , et al. Technical description, phantom accuracy, and clinical feasibility for single‐session lung radiosurgery using robotic image‐guided real‐time respiratory tumor tracking. Technol Cancer Res Treat. 2007;6(4):321‐328. 17668940 10.1177/153303460700600409

[22] Inoue M , Okawa K , Taguchi J , et al. Factors affecting the accuracy of respiratory tracking of the image‐guided robotic radiosurgery system. Jpn J Radiol. 2019;37(10):727‐734. 31367890 10.1007/s11604-019-00859-7

[23] Ziegler M , Lettmaier S , Fietkau R , et al. Performance of makerless tracking for gimbaled dynamic tumor tracking. Z Med Phys. 2020;30(2):96‐103. 31780095 10.1016/j.zemedi.2019.10.003

[24] Dieterich S , Cavedon C , Chuang CF , et al. Report of AAPM TG 135: quality assurance for robotic radiosurgery. Med Phys. 2011;38(6):2914‐2936. 21815366 10.1118/1.3579139

[25] Seppenwoolde Y , Berbeco RI , Nishioka S , et al. Accuracy of tumor motion compensation algorithm from a robotic respiratory tracking system: a simulation study. Med Phys. 2007;34(7):2774‐2784. 17821984 10.1118/1.2739811

[26] Hoogeman M , Prévost JB , Nuyttens J , et al. Clinical accuracy of the respiratory tumor tracking system of the cyberknife: assessment by analysis of log files. Int J Radiat Oncol Biol Phys. 2009;74(1):297‐303. 19362249 10.1016/j.ijrobp.2008.12.041

[27] Sumida I , Shiomi H , Higashinaka N , et al. Evaluation of tracking accuracy of the CyberKnife system using a webcam and printed calibrated grid. J Appl Clin Med Phys. 2016;17(2):74‐84. 27074474 10.1120/jacmp.v17i2.5914 PMC5875552

[28] Akino Y , Sumida I , Shiomi H , et al. Evaluation of the accuracy of the CyberKnife Synchrony™ Respiratory Tracking System using a plastic scintillator. Med Phys. 2018. doi:10.1002/mp.13028 29858498

[29] Nakayama M , Nishimura H , Mayahara H , et al. Clinical log data analysis for assessing the accuracy of the CyberKnife fiducial‐free lung tumor tracking system. Pract Radiat Oncol. 2018;8(2):e63‐e70. 29329997 10.1016/j.prro.2017.10.014

[30] Akino Y , Shiomi H , Sumida I , et al. Impacts of respiratory phase shifts on motion‐tracking accuracy of the CyberKnife Synchrony™ Respiratory Tracking System. Med Phys. 2019;46(9):3757‐3766. 30943311 10.1002/mp.13523

[31] Pepin EW , Wu H , Zhang Y , et al. Correlation and prediction uncertainties in the cyberknife synchrony respiratory tracking system. Med Phys. 2011;38(7):4036‐4044. 21859002 10.1118/1.3596527 PMC3139505

[32] Ricotti R , Seregni M , Ciardo D , et al. Evaluation of target coverage and margins adequacy during CyberKnife Lung Optimized Treatment. Med Phys. 2018;45(4):1360‐1368. 29431863 10.1002/mp.12804

[33] Case RB , Moseley DJ , Sonke JJ , et al. Interfraction and intrafraction changes in amplitude of breathing motion in stereotactic liver radiotherapy. Int J Radiat Oncol Biol Phys. 2010;77(3):918‐925. 20207501 10.1016/j.ijrobp.2009.09.008

[34] Cvek J , Knybel L , Molenda L , et al. A single reference measurement can predict liver tumor motion during respiration. Rep Pract Oncol Radiother. 2016;21(3):278‐283. 27601962 10.1016/j.rpor.2015.11.003 PMC5002026

[35] Liang Z , Liu H , Xue J , et al. Evaluation of the intra‐ and interfractional tumor motion and variability by fiducial‐based real‐time tracking in liver stereotactic body radiation therapy. J Appl Clin Med Phys. 2018;19(3):94‐100. 10.1002/acm2.12292 PMC5978939 29493095

[36] Liang Z , Zhou Q , Yang J , et al. Artificial intelligence‐based framework in evaluating intrafraction motion for liver cancer robotic stereotactic body radiation therapy with fiducial tracking. Med Phys. 2020. doi:10.1002/mp.14501 32996131

[37] Sothmann T , Blanck O , Poels K , et al. Real time tracking in liver SBRT: comparison of CyberKnife and Vero by planning structure‐based gamma‐evaluation and dose‐area‐histograms. Phys Med Biol. 2016;61(4):1677‐1691. 26836488 10.1088/0031-9155/61/4/1677

