# Supplementary material for: Novel utilization and quantification of Xsight diaphragm tracking for respiratory motion compensation in Cyberknife Synchrony treatment of liver tumors
Source: J Appl Clin Med Phys. 2024 Apr 15;25(7):e14341. doi: 10.1002/acm2.14341 (PMC11244677; doi:10.1002/acm2.14341)
Supplement: Supplementary file 1 — Appendix 1a: The tumor anatomical locations for patient1. Appendix 1b: The tumor anatomical locations for patient2. Appendix 1c: The tumor anatomical locations for patient3. Appendix 1d: The tumor anatomical locations for patient4. Appendix 1e: The tumor anatomical locations for patient5. Appendix 1f: The tumor anatomical locations for patient6. Appendix 1g: The tumor anatomical locations for patient7. Appendix 1h: The tumor anatomical locations for patient8. Appendix 1i: The tumor anatomical locations for patient9. Appendix 1j: The tumor anatomical locations for patient10. Appendix 1k: The tumor anatomical locations for patient11. [file ACM2-25-e14341-s002.zip › Appendix_1/acm214341-sup-0001-Appendix1.docx]

Appendix 1: The approximate distribution of tumor anatomical locations for all patients (XDTS: 1a-1h, FTTS: 1i-1k): 3D-view (upper left), coronal slice (upper right), DRRs generated from XDT. The blue and red regions represented the tracking tumor volume (TTV) and PTV (planning target position).
