# Supplementary figures and images for: Novel utilization and quantification of Xsight diaphragm tracking for respiratory motion compensation in Cyberknife Synchrony treatment of liver tumors
Source: J Appl Clin Med Phys. 2024 Apr 15;25(7):e14341. doi: 10.1002/acm2.14341 (PMC11244677; doi:10.1002/acm2.14341)

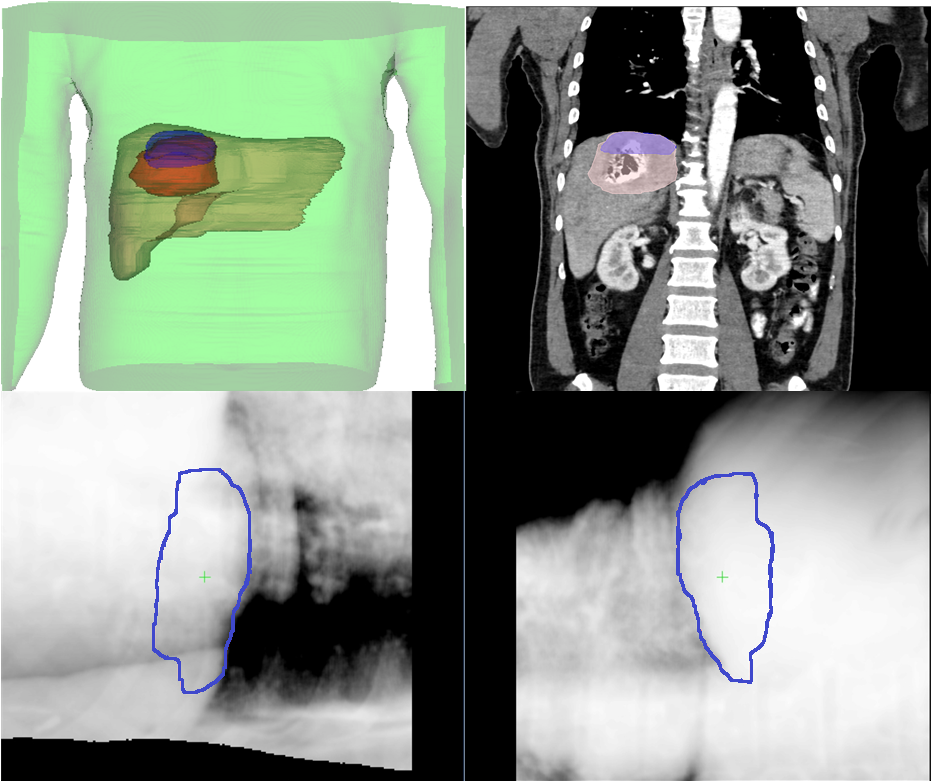

Supplement: Supplementary file 1 — Appendix 1a: The tumor anatomical locations for patient1. Appendix 1b: The tumor anatomical locations for patient2. Appendix 1c: The tumor anatomical locations for patient3. Appendix 1d: The tumor anatomical locations for patient4. Appendix 1e: The tumor anatomical locations for patient5. Appendix 1f: The tumor anatomical locations for patient6. Appendix 1g: The tumor anatomical locations for patient7. Appendix 1h: The tumor anatomical locations for patient8. Appendix 1i: The tumor anatomical locations for patient9. Appendix 1j: The tumor anatomical locations for patient10. Appendix 1k: The tumor anatomical locations for patient11. [file ACM2-25-e14341-s002.zip › Appendix_1/2023-06218-f09-z-4c.tif]

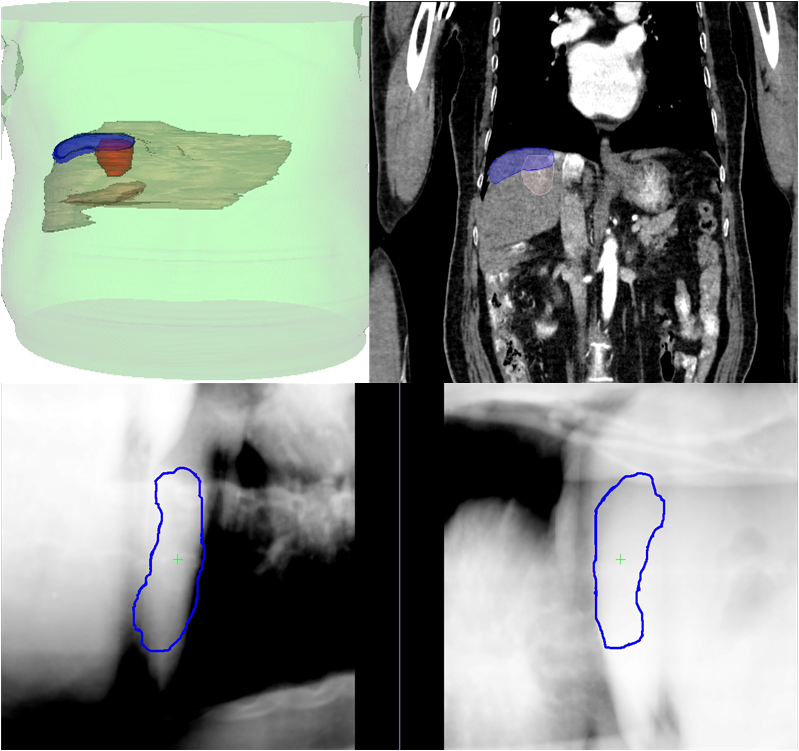

Supplement: Supplementary file 1 — Appendix 1a: The tumor anatomical locations for patient1. Appendix 1b: The tumor anatomical locations for patient2. Appendix 1c: The tumor anatomical locations for patient3. Appendix 1d: The tumor anatomical locations for patient4. Appendix 1e: The tumor anatomical locations for patient5. Appendix 1f: The tumor anatomical locations for patient6. Appendix 1g: The tumor anatomical locations for patient7. Appendix 1h: The tumor anatomical locations for patient8. Appendix 1i: The tumor anatomical locations for patient9. Appendix 1j: The tumor anatomical locations for patient10. Appendix 1k: The tumor anatomical locations for patient11. [file ACM2-25-e14341-s002.zip › Appendix_1/2023-06218-f10-z-4c.tif]

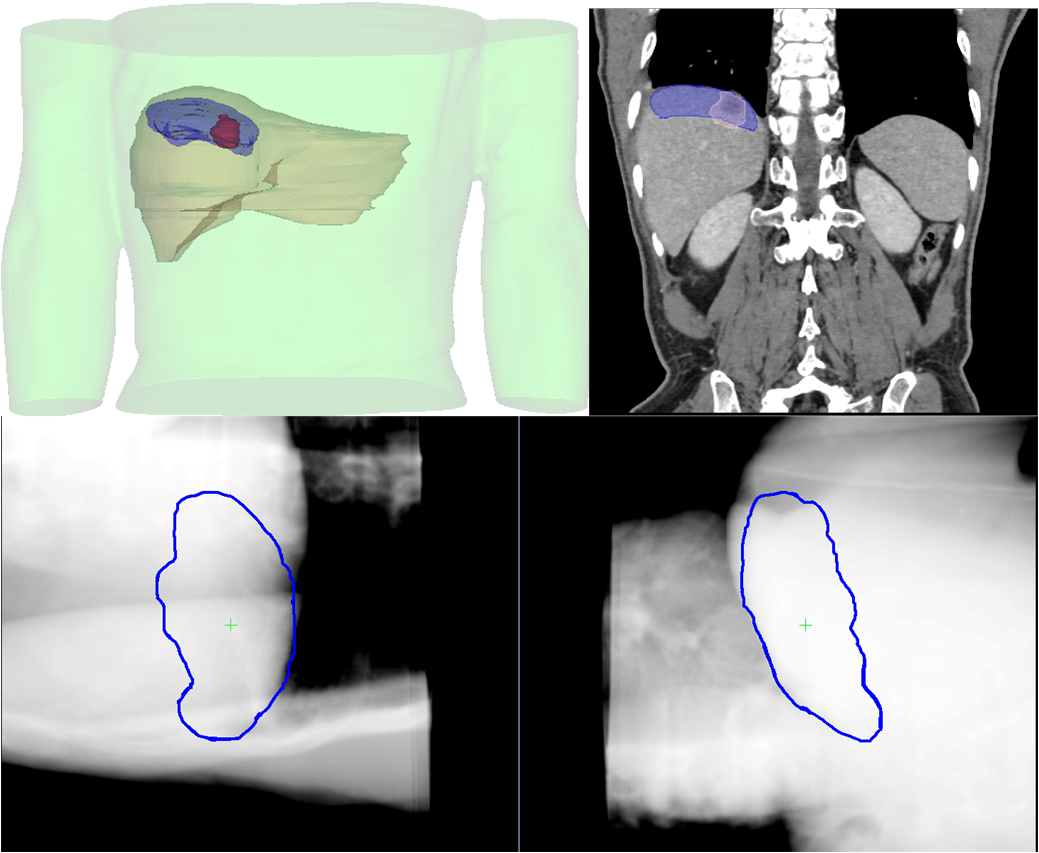

Supplement: Supplementary file 1 — Appendix 1a: The tumor anatomical locations for patient1. Appendix 1b: The tumor anatomical locations for patient2. Appendix 1c: The tumor anatomical locations for patient3. Appendix 1d: The tumor anatomical locations for patient4. Appendix 1e: The tumor anatomical locations for patient5. Appendix 1f: The tumor anatomical locations for patient6. Appendix 1g: The tumor anatomical locations for patient7. Appendix 1h: The tumor anatomical locations for patient8. Appendix 1i: The tumor anatomical locations for patient9. Appendix 1j: The tumor anatomical locations for patient10. Appendix 1k: The tumor anatomical locations for patient11. [file ACM2-25-e14341-s002.zip › Appendix_1/2023-06218-f11-z-4c.tif]

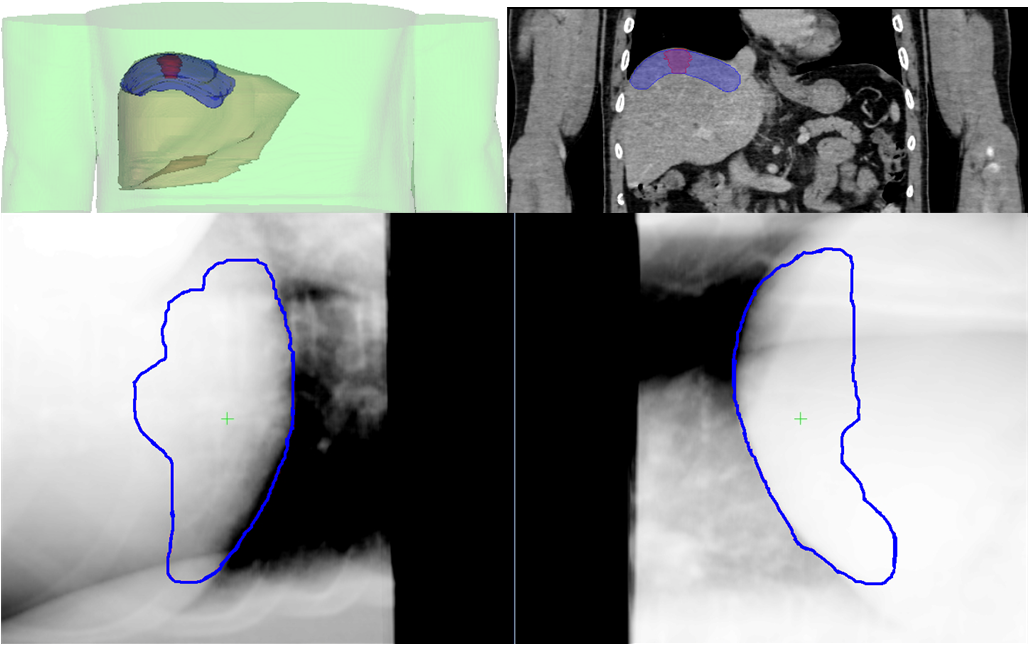

Supplement: Supplementary file 1 — Appendix 1a: The tumor anatomical locations for patient1. Appendix 1b: The tumor anatomical locations for patient2. Appendix 1c: The tumor anatomical locations for patient3. Appendix 1d: The tumor anatomical locations for patient4. Appendix 1e: The tumor anatomical locations for patient5. Appendix 1f: The tumor anatomical locations for patient6. Appendix 1g: The tumor anatomical locations for patient7. Appendix 1h: The tumor anatomical locations for patient8. Appendix 1i: The tumor anatomical locations for patient9. Appendix 1j: The tumor anatomical locations for patient10. Appendix 1k: The tumor anatomical locations for patient11. [file ACM2-25-e14341-s002.zip › Appendix_1/2023-06218-f12-z-4c.tif]

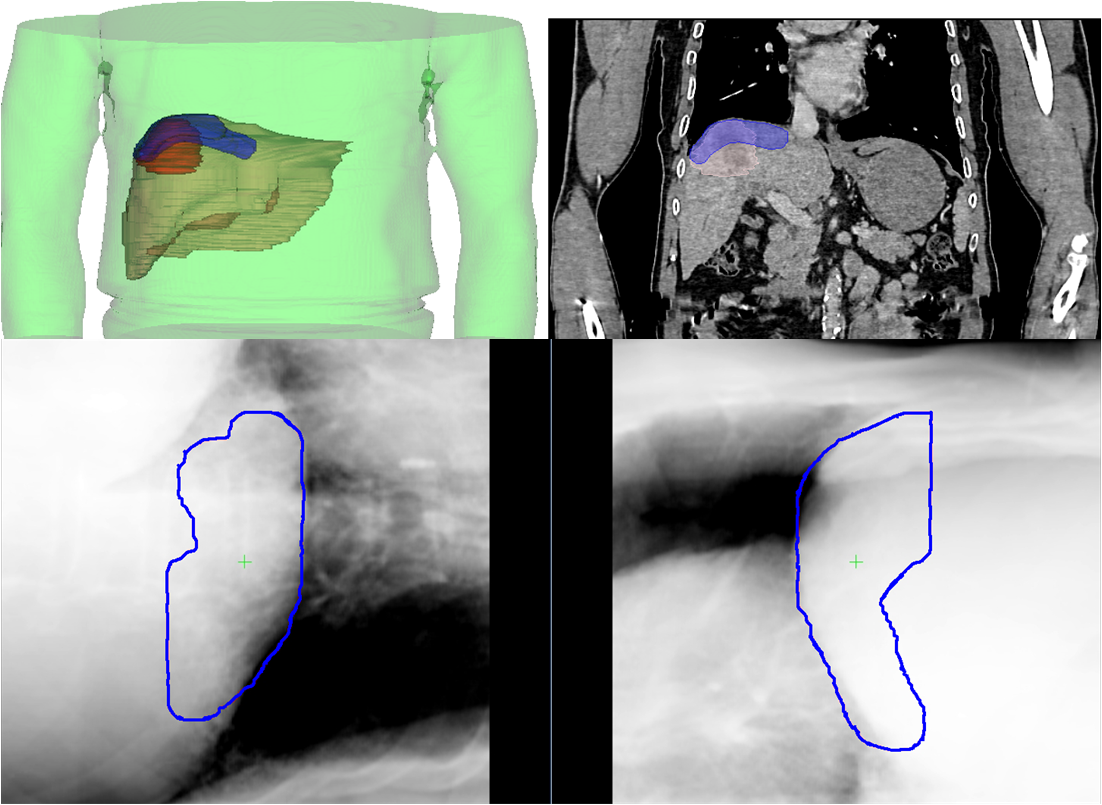

Supplement: Supplementary file 1 — Appendix 1a: The tumor anatomical locations for patient1. Appendix 1b: The tumor anatomical locations for patient2. Appendix 1c: The tumor anatomical locations for patient3. Appendix 1d: The tumor anatomical locations for patient4. Appendix 1e: The tumor anatomical locations for patient5. Appendix 1f: The tumor anatomical locations for patient6. Appendix 1g: The tumor anatomical locations for patient7. Appendix 1h: The tumor anatomical locations for patient8. Appendix 1i: The tumor anatomical locations for patient9. Appendix 1j: The tumor anatomical locations for patient10. Appendix 1k: The tumor anatomical locations for patient11. [file ACM2-25-e14341-s002.zip › Appendix_1/2023-06218-f13-z-4c.tif]

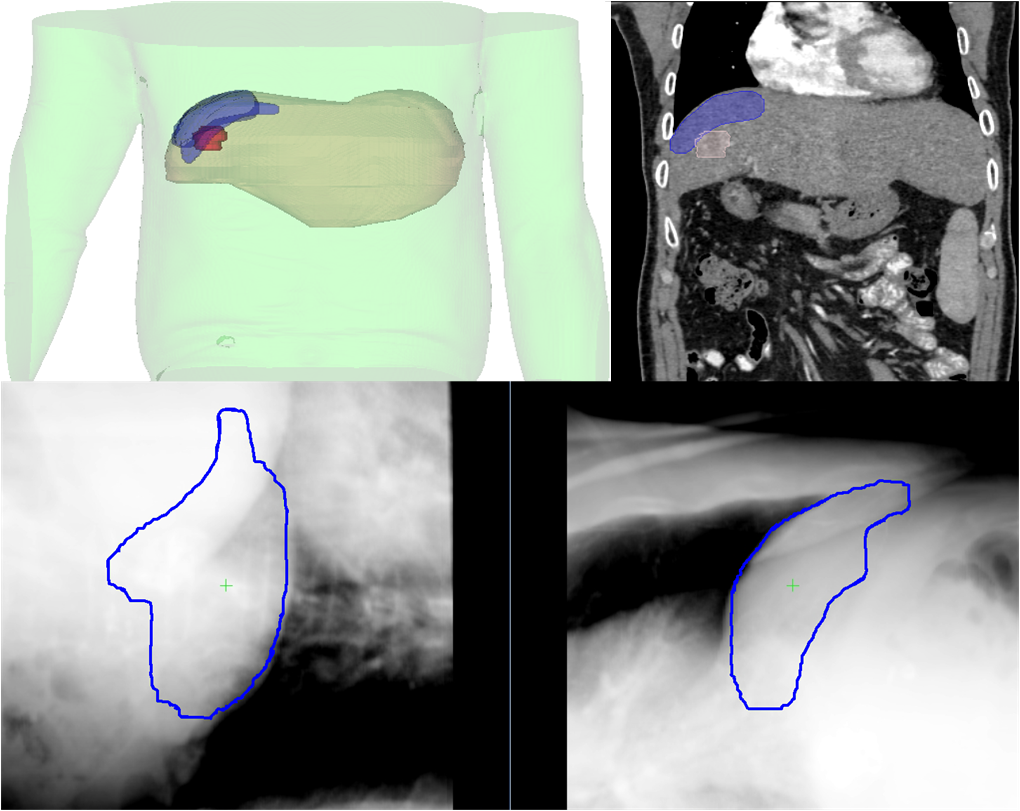

Supplement: Supplementary file 1 — Appendix 1a: The tumor anatomical locations for patient1. Appendix 1b: The tumor anatomical locations for patient2. Appendix 1c: The tumor anatomical locations for patient3. Appendix 1d: The tumor anatomical locations for patient4. Appendix 1e: The tumor anatomical locations for patient5. Appendix 1f: The tumor anatomical locations for patient6. Appendix 1g: The tumor anatomical locations for patient7. Appendix 1h: The tumor anatomical locations for patient8. Appendix 1i: The tumor anatomical locations for patient9. Appendix 1j: The tumor anatomical locations for patient10. Appendix 1k: The tumor anatomical locations for patient11. [file ACM2-25-e14341-s002.zip › Appendix_1/2023-06218-f14-z-4c.tif]

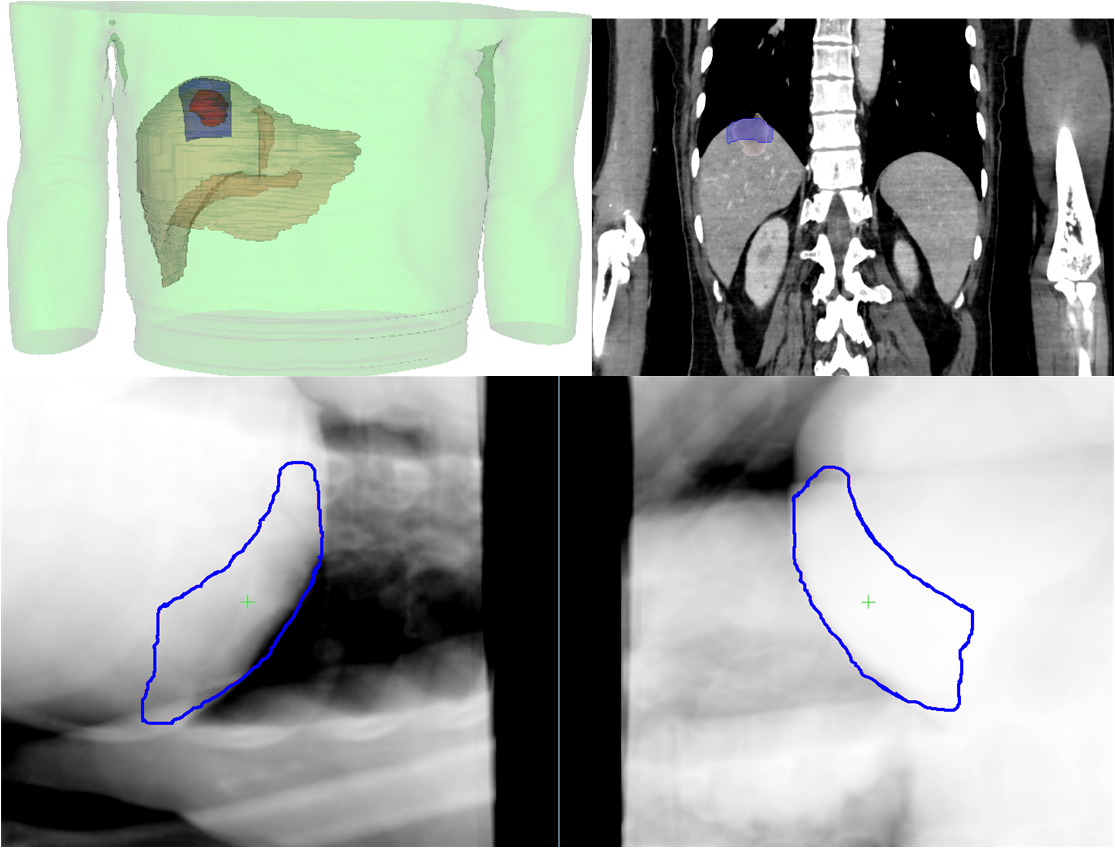

Supplement: Supplementary file 1 — Appendix 1a: The tumor anatomical locations for patient1. Appendix 1b: The tumor anatomical locations for patient2. Appendix 1c: The tumor anatomical locations for patient3. Appendix 1d: The tumor anatomical locations for patient4. Appendix 1e: The tumor anatomical locations for patient5. Appendix 1f: The tumor anatomical locations for patient6. Appendix 1g: The tumor anatomical locations for patient7. Appendix 1h: The tumor anatomical locations for patient8. Appendix 1i: The tumor anatomical locations for patient9. Appendix 1j: The tumor anatomical locations for patient10. Appendix 1k: The tumor anatomical locations for patient11. [file ACM2-25-e14341-s002.zip › Appendix_1/2023-06218-f15-z-4c.tif]

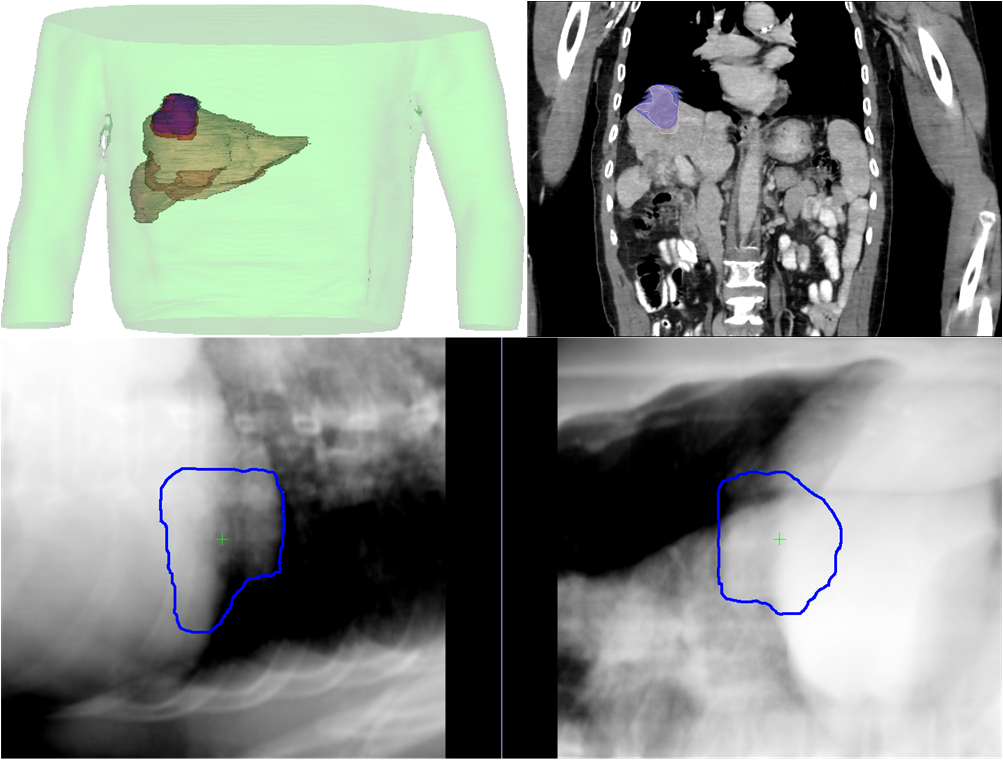

Supplement: Supplementary file 1 — Appendix 1a: The tumor anatomical locations for patient1. Appendix 1b: The tumor anatomical locations for patient2. Appendix 1c: The tumor anatomical locations for patient3. Appendix 1d: The tumor anatomical locations for patient4. Appendix 1e: The tumor anatomical locations for patient5. Appendix 1f: The tumor anatomical locations for patient6. Appendix 1g: The tumor anatomical locations for patient7. Appendix 1h: The tumor anatomical locations for patient8. Appendix 1i: The tumor anatomical locations for patient9. Appendix 1j: The tumor anatomical locations for patient10. Appendix 1k: The tumor anatomical locations for patient11. [file ACM2-25-e14341-s002.zip › Appendix_1/2023-06218-f16-z-4c.tif]

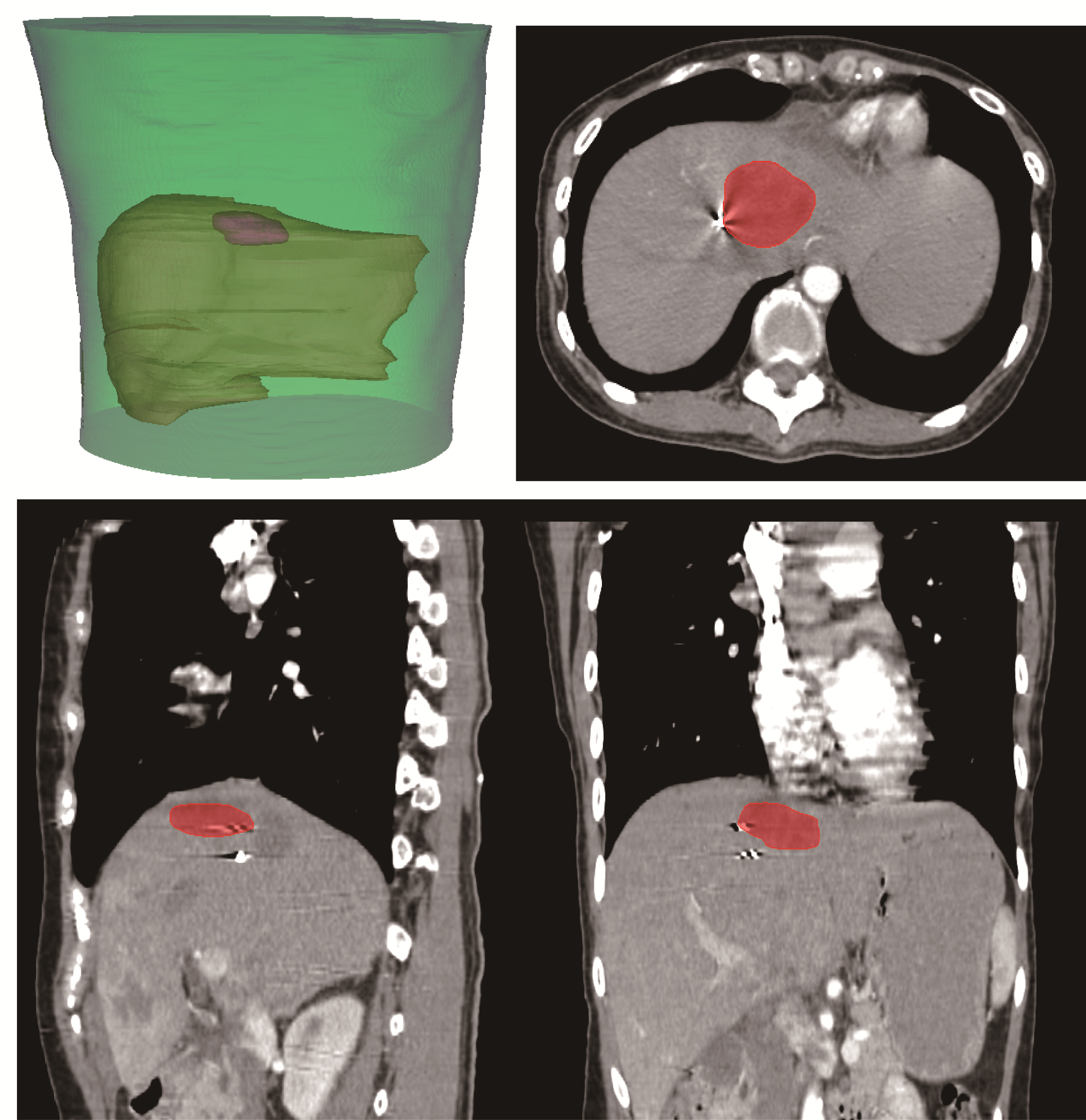

Supplement: Supplementary file 1 — Appendix 1a: The tumor anatomical locations for patient1. Appendix 1b: The tumor anatomical locations for patient2. Appendix 1c: The tumor anatomical locations for patient3. Appendix 1d: The tumor anatomical locations for patient4. Appendix 1e: The tumor anatomical locations for patient5. Appendix 1f: The tumor anatomical locations for patient6. Appendix 1g: The tumor anatomical locations for patient7. Appendix 1h: The tumor anatomical locations for patient8. Appendix 1i: The tumor anatomical locations for patient9. Appendix 1j: The tumor anatomical locations for patient10. Appendix 1k: The tumor anatomical locations for patient11. [file ACM2-25-e14341-s002.zip › Appendix_1/2023-06218-f17-z-4c.png]

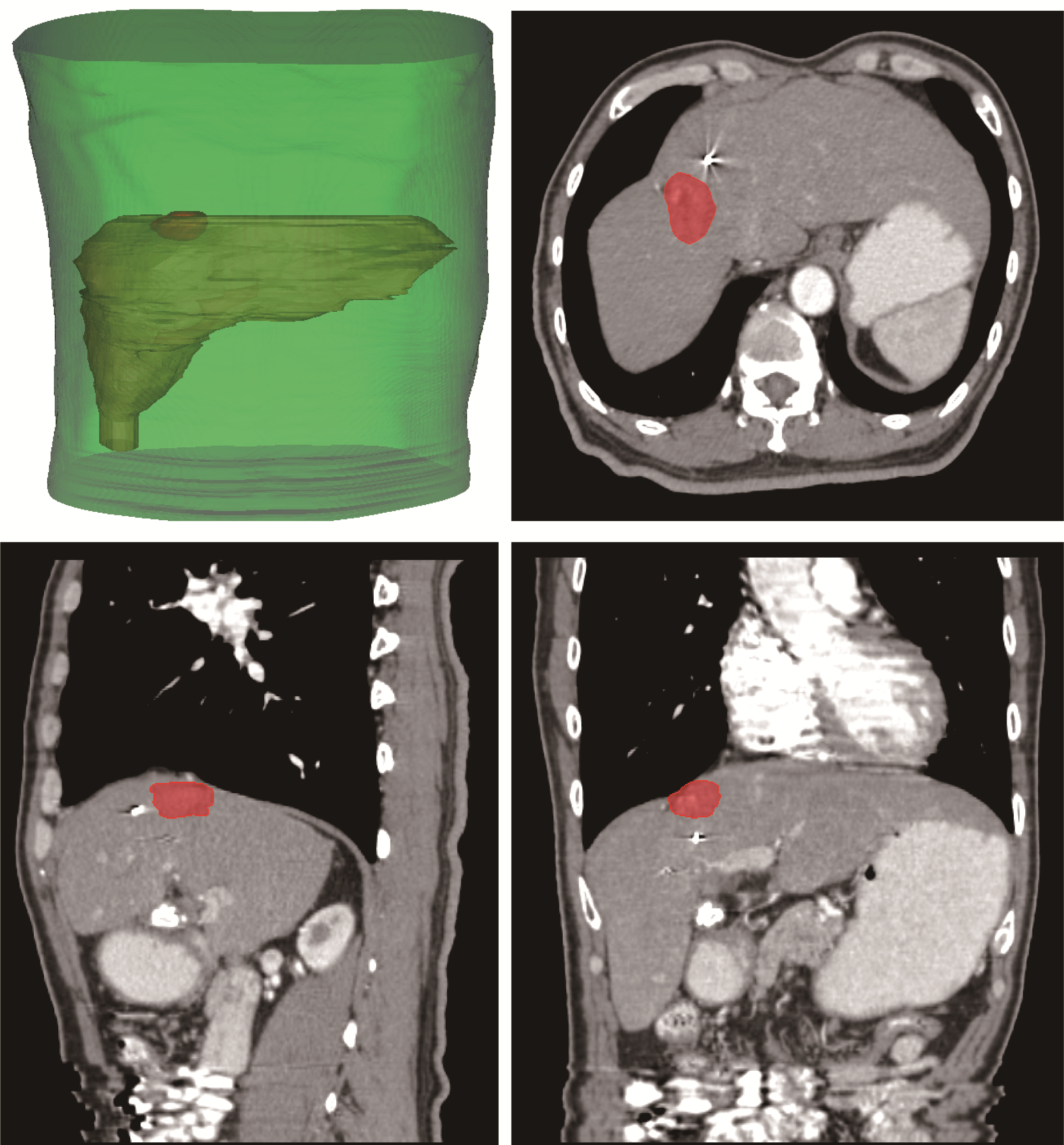

Supplement: Supplementary file 1 — Appendix 1a: The tumor anatomical locations for patient1. Appendix 1b: The tumor anatomical locations for patient2. Appendix 1c: The tumor anatomical locations for patient3. Appendix 1d: The tumor anatomical locations for patient4. Appendix 1e: The tumor anatomical locations for patient5. Appendix 1f: The tumor anatomical locations for patient6. Appendix 1g: The tumor anatomical locations for patient7. Appendix 1h: The tumor anatomical locations for patient8. Appendix 1i: The tumor anatomical locations for patient9. Appendix 1j: The tumor anatomical locations for patient10. Appendix 1k: The tumor anatomical locations for patient11. [file ACM2-25-e14341-s002.zip › Appendix_1/2023-06218-f18-z-4c.png]

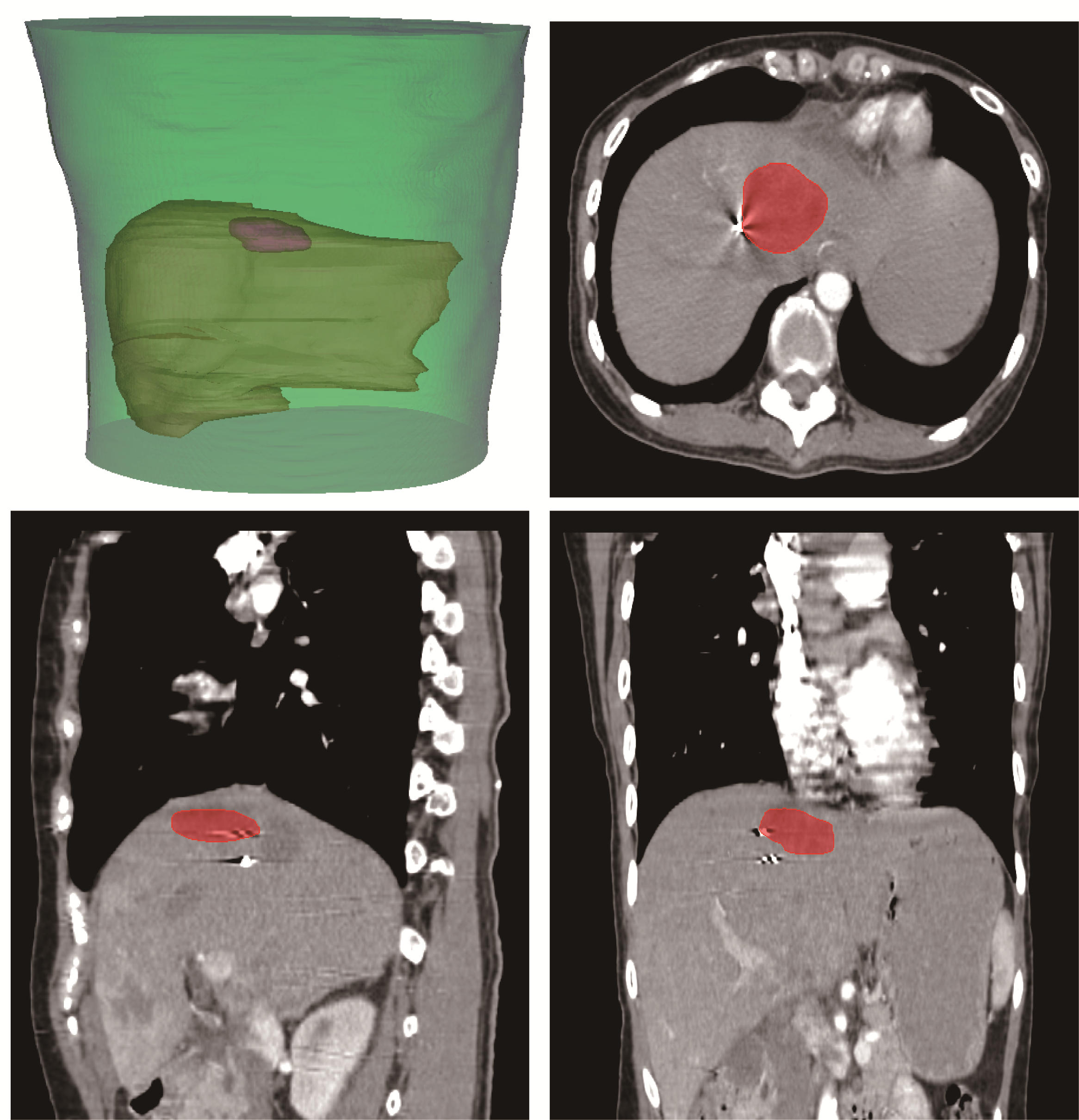

Supplement: Supplementary file 1 — Appendix 1a: The tumor anatomical locations for patient1. Appendix 1b: The tumor anatomical locations for patient2. Appendix 1c: The tumor anatomical locations for patient3. Appendix 1d: The tumor anatomical locations for patient4. Appendix 1e: The tumor anatomical locations for patient5. Appendix 1f: The tumor anatomical locations for patient6. Appendix 1g: The tumor anatomical locations for patient7. Appendix 1h: The tumor anatomical locations for patient8. Appendix 1i: The tumor anatomical locations for patient9. Appendix 1j: The tumor anatomical locations for patient10. Appendix 1k: The tumor anatomical locations for patient11. [file ACM2-25-e14341-s002.zip › Appendix_1/2023-06218-f19-z-4c.png]
