# Supplementary material for: Novel utilization and quantification of Xsight diaphragm tracking for respiratory motion compensation in Cyberknife Synchrony treatment of liver tumors
Source: J Appl Clin Med Phys. 2024 Apr 15;25(7):e14341. doi: 10.1002/acm2.14341 (PMC11244677; doi:10.1002/acm2.14341)
Supplement: Supplementary file 2 — Supporting Information [file ACM2-25-e14341-s001.zip › Appendix_2/acm214341-sup-0002-Appendix2.docx]

Appendix 2: Comparison of FTTS and XDTS regarding peak-to-peak amplitude, baseline shifts, SD of center phase, correlation error and prediction error in all directions.
